# Parental Acceptance of Fetal Tissue Donation

**DOI:** 10.1001/jamanetworkopen.2024.44238

**Published:** 2024-11-08

**Authors:** Yousif Dawood, Maurice J. B. van den Hoff, Anita C. J. Ravelli, Bernadette S. de Bakker, Eva Pajkrt

**Affiliations:** 1 Department of Medical Biology, Amsterdam University Medical Center, University of Amsterdam, Amsterdam, the Netherlands; 2 Department of Obstetrics and Gynaecology, Amsterdam University Medical Center, University of Amsterdam, Amsterdam, the Netherlands; 3 Amsterdam Reproduction and Development Research Institute, Amsterdam, the Netherlands; 4 Department of Medical Informatics, Amsterdam University Medical Center, University of Amsterdam, Amsterdam, the Netherlands; 5 Department of Paediatric Surgery, Erasmus MC–Sophia Children’s Hospital, University Medical Center Rotterdam, Rotterdam, the Netherlands

## Abstract

**Importance:**

Human fetal tissue is essential for biomedical research, providing unparalleled insights into human development and disease.

**Objective:**

To assess changes in parental decisions to donate fetal tissue following termination of pregnancy after the introduction of the Dutch Fetal Biobank (DFB) and to identify factors associated with consent to donate.

**Design, Setting, and Participants:**

This cohort study collected data from all individuals assigned female at birth (hereafter referred to as participants) who underwent a termination of pregnancy at the Amsterdam University Medical Center from January 1, 2008, to December 31, 2022. No exclusion criteria were applied.

**Exposure:**

Introduction of the DFB on September 1, 2017, which offers the option to donate fetal tissue after pregnancy termination.

**Main Outcome and Measure:**

Rates of fetal tissue donation before and after DFB introduction and associations between patient characteristics (maternal age, race and ethnicity, socioeconomic status, gestational age, and reason for termination) and consent to donation were assessed using multivariable binary logistic regression modeling.

**Results:**

Of a total of 1272 participants (mean [SD] age, 33.0 [5.4] years), 576 (45.3%) were nulliparous. The mean (SD) gestational age at termination was 18 weeks 3 days (26 days), and reasons for termination were primarily because of structural defects (567 participants [44.6%]), with only a small portion (58 participants [4.6%]) terminating for social reasons (eg, unwanted or unplanned pregnancy). Fetal tissue donations increased from 1.2% (8 donations among 663 terminations) before the DFB introduction to 21.7% (132 donations among 609 terminations) after its introduction. This rise was primarily due to a shift from collective cremation to donation, while individual cremation or burial rates remained stable. The consent rate was 30.3% (132 of 436) for participants informed about the donation option. No significant demographic differences were found between participants who consented and those who did not, except for gestational age; consent rates decreased with advanced gestational age (odds ratio per week, 0.88; 95% CI, 0.83-0.94). Higher consent rates were observed for terminations due to social reasons (odds ratio, 3.56; 95% CI, 1.40-9.10).

**Conclusions and Relevance:**

These findings suggest that a substantial proportion of individuals may be willing to donate fetal tissue for biomedical research after pregnancy termination. Integrating donation options in posttermination counseling respects patient autonomy and could ethically increase tissue availability for research. Expanding biobanks and fostering international collaboration is crucial for standardizing practices and ensuring equitable research benefits.

## Key Points

**Question:**

How have parental decisions to donate fetal tissue following termination of pregnancy changed after the introduction of the Dutch Fetal Biobank (DFB), and what factors are associated with consent to donation?

**Findings:**

In this cohort study of 1272 participants, fetal tissue donations significantly increased from 1.2% to 21.7% after the DFB’s introduction, with 30.3% of informed participants consenting to donate. Consent rates were associated with gestational age and reasons for termination, with higher consent rates for social reasons for terminations and lower rates as gestational age advanced.

**Meaning:**

These findings highlight the importance of offering donation options during posttermination counseling to respect patient autonomy and ethically enhance the availability of critical research tissues.

## Teaser

This cohort study assesses changes in parental decisions to donate fetal tissue after pregnancy termination since the introduction of the Dutch Fetal Biobank and factors associated with consent.

## Introduction

Human fetal tissue is a critical resource in biomedical research, providing insights into development and disease that are unattainable through other (animal) models.^1^ Human fetal tissue, as defined by the National Institutes of Health, is tissue or cells collected from a deceased human embryo or fetus following a spontaneous or induced abortion.^1^ This type of tissue is instrumental for research that has led to millions of lives saved, especially in vaccine development and production.^2,3^ The unique proliferative and differentiation capacity of cells derived from fetal tissue compared with adult tissue has been a cornerstone in research since the 1930s.^2^ Human fetal tissue has also played a vital role in the validation and optimization of protocols for generating specialized cells or tissues from stem cells, facilitating their use in basic science and regenerative medicine.

Historically, access to human embryonic and fetal material for biomedical research has been limited. Most of our understanding of human development has been inferred from animal and in vitro models constrained by interspecies differences. The scarcity of human fetal tissue is partly due to strict legislation and complex requirements for collecting and storing this material. In response to this scarcity, the Dutch Fetal Biobank (DFB) was initiated on September 1, 2017, at the Amsterdam University Medical Center (UMC) to facilitate access to embryonic and fetal samples until 24 weeks’ gestational age,^4^ marking an important step in making fetal tissue available for research and enhancing our understanding of human development. Since its inception, the DFB has supported several key research projects, including studies on the development of the human spleen, tracheal development, and maturation of the human fetal intestinal epithelial barrier.^5,6,7^

Despite its substantial contributions, the use of human fetal tissue in biomedical research remains a topic of considerable public debate, largely as a result of its deeply rooted history in the ethically and politically complex discussion around abortion and the moral status of the fetus.^3,8^ Discussions surrounding regulations and protocols for fetal tissue donation have been notably controversial. While scientists, politicians, and religious groups have prominently voiced their opinions in these debates, the perspectives of parents who are directly faced with the choice to cremate, bury, or donate to science their embryo or fetal tissue have been almost absent.^8,9^

In the Netherlands, the shift in parental choice toward fetal tissue donation after the introduction of the DFB may offer valuable insights. These insights not only contribute to our understanding of the willingness of parents to donate but also may have implications for policy making and practice. Previous studies have suggested that the availability of biobanking options may substantially influence donation decisions and that biobanks play a crucial role in facilitating research, enhancing public trust, and ensuring informed consent, which are critical in public engagement and willingness to participate,^10,11,12,13^ but the extent to which this holds true in the context of fetal tissue donation remains underexplored. Building on these findings, the aim of this study was to evaluate the association between the introduction of the DFB and parental consent rates for fetal tissue donation.

## Methods

### Study Design and Participants

This cohort study was conducted at the Amsterdam UMC, a tertiary referral center serving the provinces of North Holland and Flevoland. We used data from all individuals assigned female at birth (hereafter referred to as participants) who underwent a termination of pregnancy between January 1, 2008, and December 31, 2022, with no exclusion criteria applied. The study was deemed nonhuman participant research by the institutional review board of Amsterdam UMC with a waiver of informed consent. This study adhered to the Strengthening the Reporting of Observational Studies in Epidemiology (STROBE) guidelines for cohort studies.

### Participant Characteristics

Participant age was measured in years. Parity was categorized as nulliparous, primiparous, and multiparous. Participant race and ethnicity, as reported in the electronic health record, was categorized as Asian (eg, Indian, Chinese); Black (eg, African, Caribbean); White (eg, European); and other, non-Western (eg, Moroccan, Turkish) to ensure that the study’s finding are more generalizable and relevant to a broader population rather than being limited to a specific group. Data on history of previous termination of pregnancy and spontaneous abortion were also extracted.

Socioeconomic status (SES) was determined using the participant’s 4-digit postal code, with data obtained from Statistics Netherlands.^14^ The SES index is calculated by Statistics Netherlands and serves as a measure of social and economic deprivation.^14^ For the analysis, participants’ SES scores were categorized into quintiles, with the first quintile representing the least deprivation and the fifth quintile the most deprivation.

### Birth Characteristics

Pregnancy date was based on the first-trimester ultrasonography scan, and gestational age at the time of termination was measured in weeks plus days. The reasons for termination were categorized based on indication, including chromosomal anomalies, monogenetic diseases (eg, cystic fibrosis), structural defects, social reasons (eg, unwanted or unplanned pregnancy), or other (eg, severe preeclampsia, infectious disease). The posttermination decisions for disposition of remains were categorized as follows: leaving them at the hospital for collective cremation, taking them home for burial or cremation, or leaving them at the hospital for donation to science. If no decision was recorded and the outcome was not documented during routine follow-up visits after the termination of pregnancy, the decision was categorized as unknown.

### Tissue Donation

The donation, storage, and use of fetal tissue have been formalized in the Netherlands since 2001.^15^ However, prior to the establishment of the DFB, patients at Amsterdam UMC were not routinely informed about the option to donate fetal tissue. With the introduction of the DFB, protocols were revised to ensure that all patients are offered the opportunity to donate the fetus. The attending gynecologist is responsible for counseling patients at the outpatient clinic after the decision to terminate the pregnancy was made. The classification of participants as informed about fetal donation was based on any relevant documentation in their electronic health record by the attending gynecologist, resident, midwife, or nurse. In the absence of such documentation, participants were categorized as not informed about fetal donation. Additionally, the performance of a postnatal autopsy was recorded, as this procedure affects the feasibility of donating the fetus. Participants who opted for a fetal autopsy were considered ineligible to donate to the DFB.^4^

### Statistical Analysis

Participant and birth characteristics were summarized and compared across the complete cohort and 3 subgroups, including those informed about fetal donation to the DFB, those informed who did not give consent, and those informed who gave consent. Differences were tested using χ^2^ test for categorical variables and independent samples *t* test for continuous variables.

We used multivariable binary logistic regression modeling to derive odds ratios (ORs) for the association of patient characteristics (age, race and ethnicity, SES score, parity, history of spontaneous abortion, and previous termination of pregnancy) and birth characteristics (gestational age at termination and reason for termination) with the outcome of donation to the DFB. This analysis was conducted using only data from participants who were informed about the option to donate to the DFB.

To enhance the robustness of our findings, 2 sensitivity analyses were conducted. The first was broadened participant inclusion. To mitigate potential selection bias introduced by professionals’ varying practices in informing patients, data from participants not informed about the DFB donation option during its operational period were also included. This approach addresses the concern that certain groups, such as women of races and ethnicities other than White, may have been excluded from being asked about donation due to assumptions or biases held by health care professionals. By including these noninformed participants, we ensured a more accurate and inclusive assessment of the factors associated with consent to donate.

The second sensitivity analysis involved a different SES categorization from quintiles. The SES scores were analyzed using 2 alternative stratification methods to ascertain the association of SES with donation outcomes. The scores were divided into tertiles (quintile 1, quintiles 2-4 combined, and quintile 5), and dichotomously (quintiles 1-4 combined and quintile 5) to differentiate between low and high SES groups.

All analyses were performed using SPSS, version 28 software (IBM Corp). A 2-sided *P* < .05 was considered significant.

## Results

In total, 1272 participants terminated pregnancies between 2008 and 2022 at the Amsterdam UMC. Table 1 summarizes demographic and medical data for all participants. The mean (SD) age was 33.0 (5.4) years, which is higher than the general pregnancy population (mean [SD], 30.9 [4.6] years).^16^ White participants made up the majority of the cohort (967 [76.0%]), which is comparable with the national percentage of the pregnant population.^16^ The remainder of the cohort comprised 92 Asian participants (7.2%), 127 Black participants (10.0%), and 86 participants of other race and ethnicity (6.8%). Regarding parity, 576 participants (45.3%) were nulliparous. A total of 264 participants (20.8%) had previously terminated a pregnancy, and 305 (24.0%) had experienced a spontaneous abortion. The mean (SD) gestational age at termination was 18 weeks 3 days (26 days). The reason for termination was mainly structural defects (567 participants [44.6%]); only a small portion terminated their pregnancy for social reasons (58 participants [4.6%]). No significant difference in maternal age, race and ethnicity, SES, parity, history of termination of pregnancy or spontaneous abortion, and reason for termination was found between the subgroup that was informed about donation and declined donation and the subgroup that gave consent for donation (Table 1; eTable 1 in Supplement 1). However, the gestational age at termination was significantly lower in the group that gave consent for donation (mean [SD], 17 weeks 0 days [27 days]) compared with those who did not give consent (mean [SD], 18 weeks 4 days [25 days]).

**Table 1.** Participants’ Clinical and Demographic Characteristics

Characteristic	No. (%)				*P* value^a^
	Total (N = 1272)	Informed donation			
		Total informed (n = 436)	No consent for donation (n = 304)	Consent for donation (n = 132)	
Age, mean (SD), y	33.0 (5.4)	33.1 (5.4)	33.1 (5.6)	33.1 (4.9)	.85
Race and ethnicity					
Asian	92 (7.2)	28 (6.4)	19 (6.3)	9 (6.8)	.86
Black	127 (10.0)	43 (9.9)	29 (9.5)	14 (10.6)	
White	967 (76.0)	338 (77.5)	236 (77.6)	102 (77.3)	
Other, non-Western^b^	86 (6.8)	27 (6.2)	20 (6.6)	7 (5.3)	
SES score, mean (SD)	0.09 (0.23)	0.09 (0.25)	0.10 (0.25)	0.07 (0.24)	.24
Lowest SES quintile^c^	252 (19.8)	87 (20.2)	60 (19.8)	27 (21.1)	.14
Parity					
Nulliparous	576 (45.3)	219 (50.2)	145 (47.7)	74 (56.1)	.19
Primiparous	462 (36.3)	142 (32.6)	105 (34.5)	37 (28.0)	
Multiparous	234 (18.4)	75 (17.2)	54 (17.8)	21 (15.9)	
History					
Terminated pregnancy	264 (20.8)	79 (18.1)	59 (19.4)	20 (15.2)	.29
Spontaneous abortion	305 (24.0)	101 (23.2)	75 (24.7)	26 (19.7)	.26
Gestational age at termination, mean (SD)	18 wk 3 d (26 d)	18 wk 1 d (26 d)	18 wk 4 d (25 d)	17 wk 0 d (27 d)	<.001
Reason for termination					
Chromosomal anomaly	477 (37.5)	184 (42.2)	125 (41.1)	59 (44.7)	.38
Monogenetic disease	141 (11.1)	46 (10.6)	36 (11.8)	10 (7.6)	
Structural defect	567 (44.6)	165 (37.8)	125 (41.1)	40 (30.3)	
Social	58 (4.6)	30 (6.9)	12 (3.9)	18 (13.6)	
Other^d^	29 (2.3)	11 (2.5)	6 (1.4)	5 (3.8)	
Posttermination decision					
Collective cremation	581 (45.7)	116 (26.6)	115 (37.8)	1 (0.8)^e^	<.001
Home for burial or cremation	539 (42.4)	189 (43.3)	189 (62.2)	0	
Donated to science	139 (10.9)	131 (30.0)	0	131 (99.2)	
Unknown	13 (1.0)	0	0	0	

Abbreviation: SES, socioeconomic status.

^a^ Differences were tested using the χ^2^ test for categorical variables and independent samples *t* test for continuous variables. Significance was defined as *P* < .05.

^b^ Other races and ethnicities included Moroccan and Turkish.

^c^ Detailed information for all SES quintiles is available in eTable 1 in Supplement 1.

^d^ Other reasons for termination included severe preeclampsia and infectious disease.

^e^ Patient gave consent for donation; however, due to unexpected events, the fetal remains were cremated.

**Figure 1.**
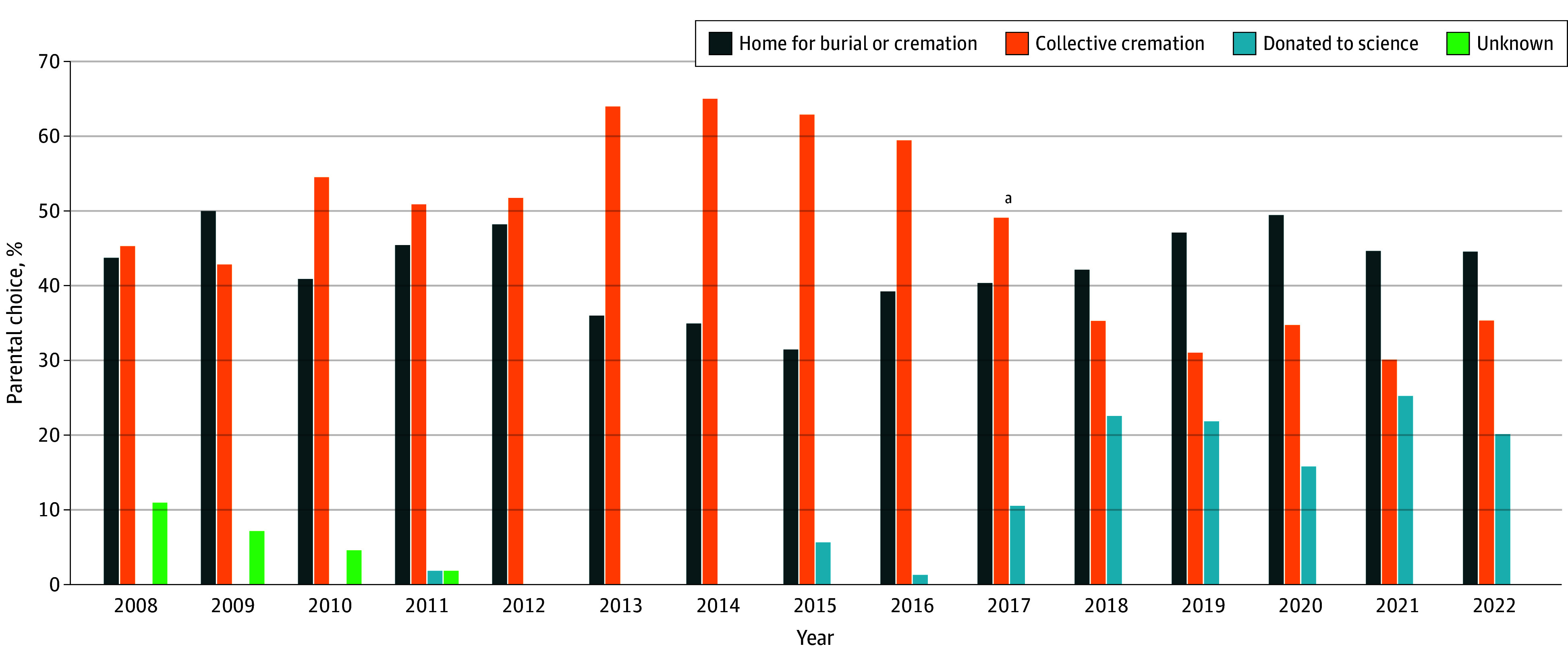
Parental Choice for Fetal Remains After Pregnancy Termination ^a^Indicates the start of the Dutch Fetal Biobank on September 1, 2017.

**Figure 2.**
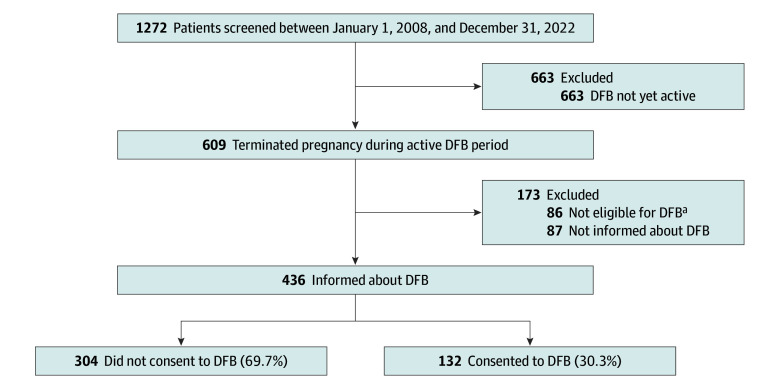
Study Flowchart ^a^Autopsy was performed after termination of pregnancy; therefore, inclusion in the Dutch Fetal Biobank (DFB) was not possible.

The rate of donations of fetal remains to science increased notably since the introduction of the DFB in 2017, from 1.2% (8 donations among 663 terminations) in 2016 to 21.7% (132 donations among 609 terminations) in 2021 (Figure 1). This increase seemed to result primarily from a change in the decision of participants who would otherwise have chosen to leave the fetal remains for collective cremation, as the number of patients choosing collective cremation decreased from 375 of 663 (56.6%) to 206 of 609 (33.8%) in the same period. At the same time, the rates of participants who decided to take the fetal remains home for burial or cremation remained fairly stable from 40.3% (267 of 663) just before the DFB’s introduction to 44.7% (272 of 609) in 2022. Furthermore, when focusing only on terminated pregnancies that were eligible for the DFB and participants who were informed about the DFB, the consent rate for donation was even higher at 30.3% (132 of 436) (Figure 2).

Among the key characteristics analyzed for their potential association with willingness to donate, including maternal age, ethnicity, SES, gestational age at termination, and reason for termination, only gestational age at termination and reason for termination were associated with the likelihood of consenting to donate (Table 2). There was a significant reduction in the odds of consenting to donate for each week increase in gestational age (OR, 0.88; 95% CI, 0.83-0.94), and a higher likelihood of consenting to donate when the pregnancy was terminated for social reasons (OR, 3.56; 95% CI, 1.40-9.10) (Table 2).

**Table 2.** Association of Maternal Age, Race and Ethnicity, SES Score, Gestational Age, and Termination Reasons With Consent for Donation

Characteristic	Consent for donation	
	OR (95% CI)	*P* value
Maternal age	1.01 (0.97-1.05)	.77
Race and ethnicity		
Asian	1.02 (0.42-2.48)	.96
Black	0.82 (0.37-1.79)	.61
White	1 [Reference]	NA
Other, non-Western^a^	0.52 (0.19-1.43)	.21
SES score, quintile		
1 (most affluent)	1 [Reference]	NA
2	0.70 (0.34-1.47)	.35
3	1.10 (0.57-2.12)	.78
4	1.17 (0.60-2.26)	.65
5 (least affluent)	1.13 (0.57-2.24)	.73
Gestational age at termination (wk)	0.88 (0.83-0.94)	<.001
Reason for termination		
Chromosomal anomaly	1 [Reference]	NA
Monogenetic disease	0.68 (0.31-1.50)	.34
Structural defect	0.89 (0.54-1.47)	.64
Social	3.56 (1.40-9.10)	.008
Other^b^	3.07 (0.78-12.05)	.11

Abbreviations: OR, odds ratio; NA, not applicable; SES, socioeconomic status.

^a^ Other races and ethnicities included Moroccan and Turkish.

^b^ Other reasons for termination included severe preeclampsia and infectious disease.

The first sensitivity analysis, which broadened participant inclusion, added 87 patients for a total of 524. The second analysis assessed SES using 2 alternative stratification methods, which included dividing SES into tertiles (eTable 2 in Supplement 1) and using a dichotomous approach (eTable 3 in Supplement 1). Importantly, neither sensitivity analysis altered the original findings, and the associations with the likelihood of consenting to donate remained for gestational age at termination and termination for social reasons (eTables 4-6 in Supplement 1).

## Discussion

Although human fetal tissue is considered an invaluable resource in biomedical research, no study to our knowledge has explored the shift in posttermination decision-making and the factors associated with a patient’s decision to donate fetal tissue from their own perspective.

Our findings have several implications for ongoing efforts to increase fetal tissue availability for biomedical research. First, the consideration of including the option for human fetal tissue donation as a part of the counseling process after termination is crucial, especially given that approximately 1 in 3 patients (30.3%) consented to donate when presented with the choice. This finding suggests that a substantial proportion of individuals may be willing to contribute to biomedical research and, by extension, may experience a sense of solace or purpose in the knowledge that their donation may lead to medical advancements. Offering this option, therefore, fulfills an ethical obligation for the medical community to inform and provide individuals with a choice that may align with their values and preferences as an alternative to burial or cremation. Furthermore, our group has previously shown that fetal donation to scientific research is not associated with decreased patient psychological well-being after termination.^17^ It is imperative that the option to donate is presented after the decision to terminate the pregnancy has been made and without pressure, ensuring that consent is voluntary and informed and respects the patient’s autonomy. By offering the option for donation, health care professionals could honor the preferences of patients who see value in contributing to research, thereby not only respecting patient autonomy but also ethically enhancing the availability of fetal tissues critical for advancing medical science.

Second, the significant increase in donations following the introduction of the DFB underscores the importance of providing patients who decided to terminate their pregnancy the option to donate the fetal remains. This finding aligns with international experiences from other types of biobanks, such as those collecting adult tissues, organs, and genetic material, where providing clear information about the benefits of donation often leads to increased willingness to participate.^18,19^ For instance, biobanking initiatives in the US and UK have shown that a robust infrastructure, along with strong ethical frameworks and public engagement strategies, may substantially boost participation rates.^20^ However, fetal tissue biobanking presents unique ethical and regulatory challenges compared with other types of biobanks given the sensitivities surrounding pregnancy termination and the moral status of the fetus. Enhancing international collaboration might play an important role in harmonizing practices and regulations, which are crucial for the expansion and efficacy of these biobanks amid a complex and varied regulatory landscape.^4^ Learning from the practices of established biobanks globally, particularly in terms of participant engagement, consent processes, and ethical oversight, may help refine the approach to fetal tissue biobanking, ensuring that it meets both scientific and public needs.^20^

Third, the lack of association with most characteristics studied (ie, maternal age, race and ethnicity, SES) suggests that a variety of individual factors and circumstances influence the decision to consent to donation and highlights the complexity of this choice. The association between increased gestational age and decreased likelihood of consenting to donate may reflect changing attitudes and emotional connections as the pregnancy advances. This trend is supported by evidence that there is an association with increased gestational age and the development of symptoms of complicated grief, postnatal depression, and posttraumatic stress following termination of pregnancy.^17,21,22,23^ In contrast, the increased likelihood of participants consenting to donate in cases of pregnancies terminated for social reasons may suggest a different decision-making process among this group. Given that these pregnancies are often unwanted, there may be a lesser emotional connection, which may lead to these individuals being more receptive to the societal benefits of donation. However, termination of unwanted pregnancy should not imply a targeted approach but rather offer insight into the broad demographics that fetal biobanks may expect to engage with.

### Limitations

This study provides valuable insights into the decision-making process regarding fetal tissue donation after pregnancy termination. However, it is not without limitations. Conducted within the Amsterdam UMC, the research benefits from a diverse patient population, yet the extent to which these findings can be extrapolated to other settings remains uncertain. The sociocultural and legal context of the Netherlands, which may facilitate a higher willingness to donate, is distinct and might not reflect the complexities of decision-making in regions with different health care systems, legal frameworks, and societal norms regarding abortion and biomedical research. Furthermore, while the study captures a range of demographic factors, the depth of individual motivations, ethical considerations, and emotional dynamics behind the decision of whether to donate is not fully explored. Qualitative investigations might enrich our understanding of these aspects.

## Conclusions

In this cohort study, we found that following the introduction of the DFB, there was a significant increase in fetal tissue donation after termination of pregnancy. Consent rates were associated with gestational age and reasons for termination, with higher rates for social terminations and lower rates as gestational age advanced, while maternal age, race and ethnicity, and SES showed no associations. The rise in fetal tissue donations following the introduction of the DFB highlights the biobank’s crucial role in advancing biomedical research and underscores the importance of further expanding biobanking initiatives. Integrating donation options into posttermination counseling respects patient autonomy and could ethically enhance tissue acquisition for continued medical advancement. We advocate for biobank expansion and international collaboration to standardize practices, ensuring equitable research benefits and prioritizing the well-being of donors.

## Notes

Supplement 1. **eTable 1.** Socioeconomic Status (SES) per Deprivation Quintile and Consent or Nonconsent for Donation **eTable 2.** Socioeconomic Status (SES) per Deprivation Tertile and Consent or Nonconsent for Donation **eTable 3.** Socioeconomic Status (SES) Dichotomized, Also per Consent or Nonconsent for Donation **eTable 4.** Sensitivity Analyses With Broadened Patient Inclusion **eTable 5.** Sensitivity Analyses With Socioeconomic Status Categorized in Tertiles **eTable 6.** Sensitivity Analyses With Socioeconomic Status Dichotomized

Supplement 2.
Data Sharing Statement
