# Supplementary material for: Parental Acceptance of Fetal Tissue Donation
Source: JAMA Netw Open. 2024 Nov 8;7(11):e2444238. doi: 10.1001/jamanetworkopen.2024.44238 (PMC11549655; doi:10.1001/jamanetworkopen.2024.44238)
Supplement: Supplement 1. — eTable 1. Socioeconomic Status (SES) per Deprivation Quintile and Consent or Nonconsent for Donation eTable 2. Socioeconomic Status (SES) per Deprivation Tertile and Consent or Nonconsent for Donation eTable 3. Socioeconomic Status (SES) Dichotomized, Also per Consent or Nonconsent for Donation eTable 4. Sensitivity Analyses With Broadened Patient Inclusion eTable 5. Sensitivity Analyses With Socioeconomic Status Categorized in Tertiles eTable 6. Sensitivity Analyses With Socioeconomic Status Dichotomized [file jamanetwopen-e2444238-s001.pdf]

Supplemental Online Content

Dawood Y, van den Hoff MJB, Ravelli ACJ, de Bakker BS, Pajkrt E. Parental acceptance of fetal tissue donation. *JAMA Netw Open*. 2024;7(11):e2444238.
doi:10.1001/jamanetworkopen.2024.44238

eTable 1. Socioeconomic Status (SES) per Deprivation Quintile and Consent or Nonconsent for Donation

eTable 2. Socioeconomic Status (SES) per Deprivation Tertile and Consent or Nonconsent for Donation

eTable 3. Socioeconomic Status (SES) Dichotomized, Also per Consent or Nonconsent for Donation

eTable 4. Sensitivity Analyses With Broadened Patient Inclusion

eTable 5. Sensitivity Analyses with Socioeconomic Status Categorized in Tertiles

eTable 6. Sensitivity Analyses With Socioeconomic Status Dichotomized

This supplemental material has been provided by the authors to give readers additional information about their work.

© 2024 Dawood Y et al. *JAMA Network Open.*

eTable 1. Socioeconomic Status (SES) per Deprivation Quintile and Consent or Nonconsent for Donation

	**Total**<br>**N = 1272**		**Informed about donation**<br>**N = 436**			**No consent for donation**<br>**n = 304**		**Consent for donation**<br>**n = 132**		**p-value**
**SES**	N (%)	Mean (s.d.)	N (%)	Mean (s.d.)		N (%)	Mean (s.d.)	N (%)	Mean (s.d.)	
**Overall**		.087 (.232)		.091 (.251)			.101 (.254)		.069 (.243)	0.237
Q1 (most affluent)	244 (19.4%)	.380 (.105)	94 (21.8%)	.403 (.145)		66 (21.8%)	.417 (.153)	28 (21.9%)	.371 (.122)	0.217
Q2	254 (20.2%)	.228 (.023)	73 (16.9%)	.228 (.024)		56 (18.5%)	.232 (.024)	17 (13.3%)	.217 (.023)	0.148
Q3	250 (19.9%)	.115 (.034)	90 (20.9%)	.118 (.032)		63 (20.8%)	.121 (.032)	27 (21.1%)	.111 (.032)	0.199
Q4	255 (20.3%)	-.007 (.052)	87 (20.2%)	-.007 (.051)		58 (19.1%)	-.007 (.055)	29 (22.7%)	-.008 (.044)	0.583
Q5 (most deprived)	252 (19.8%)	-.272 (.104)	87 (20.2%)	-.289 (.095)		60 (19.8%)	-.286 (.092)	27 (21.1%)	-.295 (.101)	0.141
Unknown[#]	17		5			1		4		

[#]Unknown due to missing information about postal code.

s.d.: standard deviation

© 2024 Dawood Y et al. *JAMA Network Open.*

eTable 2. Socioeconomic Status (SES) per Deprivation Tertile and Consent or Nonconsent for Donation

	**Total N = 1272**		**Informed about donation N = 436**			**No consent for donation n = 304**		**Consent for donation n = 132**		**p-value**
**Socio-economic status**	N (%)	Mean (s.d.)	N (%)	Mean (s.d.)		N (%)	Mean (s.d.)	N (%)	Mean (s.d.)	
Q1 (most affluent)	244 (19.4%)	.380 (.105)	94 (21.8%)	.403 (.145)		66 (21.8%)	.417 (.153)	28 (21.9%)	.371 (.122)	0.217
Q2-4	759 (60.5%)	.112 (.104)	250 (58.0%)	.107 (.102)		177 (58.2%)	.114 (.104)	73 (57.0%)	0.088 (.095)	0.227
Q5 (most deprived)	87 (20.2%)	-.289 (.095)	87 (20.2%)	-.289 (.095)		60 (19.8%)	-.286 (.092)	60 (19.8%)	-.295 (.101)	0.255
Unknown#	17		5			1		4		

#Unknown due to missing information about postal code.

s.d.: standard deviation

eTable 3. Socioeconomic Status (SES) Dichotomized, Also per Consent or Nonconsent for Donation

	**Total N = 1272**		**Informed about donation N = 436**			**No consent for donation n = 304**		**Consent for donation n = 132**		**p-value**
**Socio-economic status**	N (%)	Mean (s.d.)	N (%)	Mean (s.d.)		N (%)	Mean (s.d.)	N (%)	Mean (s.d.)	
Q1-4	1003 (79.9%)	.177 (.155)	344 (79.8%)	.188 (.175)		243 (80.2%)	.167 (.180)	101 (78.9%)	.167 (.164)	0.168
Q5 (most deprived)	252 (19.8%)	-.272 (.104)	87 (20.2%)	-.289 (.095)		60 (19.8%)	-.286 (.092)	60 (19.8%)	-.295 (.101)	0.255
Unknown#	17		5			1		4		

#Unknown due to missing information about postal code.

s.d.: standard deviation

© 2024 Dawood Y et al. *JAMA Network Open.*

eTable 4. Sensitivity Analyses With Broadened Patient Inclusion

Characteristics	Consent for donation		
	OR	95% CI	p-value
**Maternal age (years)**	1.010	0.971 - 1.052	0.612
**Ethnicity**			
White	*Reference*		N/a
Black	0.728	0.339 - 1.563	0.416
Asian	0.801	0.349 - 1.835	0.599
Other	0.516	0.197 - 1.350	0.178
**SES score**			
Q1 (most affluent)	*Reference*		N/a
Q2	0.707	0.347 - 1.440	0.339
Q3	1.137	0.600 - 2.157	0.693
Q4	1.091	0.576 - 2.068	0.789
Q5 (most deprived)	1.021	0.529 - 1.971	0.951
**Gestational age at termination (weeks)**	0.882	0.832 - 0.935	**<0.001**
**Reason for termination**			
Chromosomal	*Reference*		N/a
Monogenetic	0.687	0.317 - 1.487	0.340
Structural	0.800	0.492 - 1.302	0.370
Social	2.505	1.062 - 5.911	**0.036**
Other	2.115	0.631 - 7.090	0.225

Association of maternal age, ethnicity, socio-economic status scores, gestational age, and termination reasons with consent for donation.

OR: odds ratio, CI: confidence interval, Q: quintile of deprivation

© 2024 Dawood Y et al. *JAMA Network Open.*

eTable 5. Sensitivity Analyses With Socioeconomic Status Categorized in Tertiles

Characteristics	Consent for donation		
	OR	95% CI	p-value
**Maternal age (years)**	1.006	0.965 - 1.049	0.779
**Ethnicity**			
White	*Reference*		N/a
Black	0.833	0.381 - 1.821	0.647
Asian	0.931	0.394 - 2.202	0.871
Other	0.562	0.205 - 1.539	0.262
**SES score**			
Q1 (most affluent)	*Reference*		N/a
Q2-4	0.967	0.563 - 1.663	0.904
Q5 (most deprived)	1.108	0.558 - 2.197	0.770
**Gestational age at termination (weeks)**	0.884	0.831 - 0.940	**<0.001**
**Reason for termination**			
Chromosomal	*Reference*		N/a
Monogenetic	0.696	0.317 - 1.530	0.367
Structural	0.900	0.545 - 1.485	0.680
Social	3.723	1.458 - 9.505	**0.006**
Other	3.293	0.843 - 12.873	0.087

Association of maternal age, ethnicity, socio-economic status scores, gestational age, and termination reasons with consent for donation.
OR: odds ratio, CI: confidence interval, Q: quintile of deprivation

© 2024 Dawood Y et al. *JAMA Network Open.*

eTable 6. Sensitivity Analyses With Socioeconomic Status Dichotomized

Characteristics	Consent for donation		
	OR	95% CI	p-value
**Maternal age (years)**	1.006	0.965 - 1.049	0.781
**Ethnicity**			
White	*Reference*		N/a
Black	0.831	0.380 - 1.814	0.641
Asian	0.928	0.393 - 2.190	0.864
Other	0.559	0.205 - 1.526	0.256
**SES score**			
Q1-4 (most affluent)	*Reference*		N/a
Q5 (most deprived)	1.135	0.651 - 1.979	0.655
**Gestational age at termination (weeks)**	0.884	0.831 - 0.940	**<0.001**
**Reason for termination**			
Chromosomal	*Reference*		N/a
Monogenetic	0.697	0.318 - 1.531	0.369
Structural	0.900	0.545 - 1.485	0.680
Social	3.716	1.456 - 9.486	**0.006**
Other	3.288	0.840 - 12.865	0.087

Association of maternal age, ethnicity, socio-economic status scores, gestational age, and termination reasons with consent for donation.
OR: odds ratio, CI: confidence interval, Q: quintile of deprivation

© 2024 Dawood Y et al. *JAMA Network Open.*
