# Supplementary material for: Parental Acceptance of Fetal Tissue Donation
Source: JAMA Netw Open. 2024 Nov 8;7(11):e2444238. doi: 10.1001/jamanetworkopen.2024.44238 (PMC11549655; doi:10.1001/jamanetworkopen.2024.44238)
Supplement: Supplement 2. — Data Sharing Statement [file jamanetwopen-e2444238-s002.pdf]

# Data Sharing Statement

Dawood. Parental Acceptance of Fetal Tissue Donation. *JAMA Netw Open*. Published November 08, 2024. doi:10.1001/jamanetworkopen.2024.44238

**Data**
**Data available:** No
